# Job satisfaction among family nurses in Poland: A questionnaire‐based study

**DOI:** 10.1002/nop2.550

**Published:** 2020-07-09

**Authors:** Paulina Kalinowska, Ludmila Marcinowicz

**Affiliations:** ^1^ Department of Obstetrics, Gynaecology and Maternity Care Medical University of Bialystok Bialystok Poland

**Keywords:** job satisfaction, nurses, nursing, Poland

## Abstract

**Aim:**

To define the level of job satisfaction among Polish family nurses. Attempts were made to assess whether job satisfaction depends on the job location, form of employment, family structure and financial situation.

**Design:**

A cross‐sectional study was conducted among Polish family nurses who were professionally active in 2018.

**Method:**

A self‐administered questionnaire which included a standardized questionnaire “The Satisfaction with Job Scale” by A. M. Zalewska and our survey questionnaire was administered to 225 of participants (returned 220). The data were collected in 2018.

**Results:**

Our study showed that the Polish family nurses are moderately satisfied with their job. A higher level of job satisfaction was reported by those family nurses with longer job seniority, those who were working in the country, those who were owners or co‐owners of a primary healthcare unit, those who were living in a complete family or as single and those who could afford to buy what they wanted and possessed savings.

**Abbreviations**
Me: median
OIPiP: District Chamber of Nurses and Midwives
Q1: the lower quartile
Q3: the upper quartile
*SD*: standard deviation

## 1 INTRODUCTION

Professional work occupies a significant part in our life. It is satisfaction of financial and material needs, but also has social and psychological dimension. Job satisfaction has a direct impact on the individual's life, self‐esteem and fulfilment. The concept of satisfaction, despite many attempts to standardize it, is still a difficult category to standardize. The first research results on job satisfaction were published in the 1930s (Hersey, 1932; Hoppock, 1935; Mayo, 1933). The reason for interest in this topic was the consequences of job satisfaction for employees and the organization expected by researchers and practitioners.

From the 60s, job satisfaction is defined as “an emotional reaction of pleasure or resentment with an employee derives from fulfilling given tasks, functions and roles” (Bańka, 2005; Locke, 1976; Spector, 1997). Then in the 1980s, the scope of research was broadened and the studied problems deepened. This included the relationship between job satisfaction and various categories of behaviour at work. Additionally, the relationship between job satisfaction and functioning in other spheres of life and satisfaction derived from it was determined (Brief & Roberson, 1989; Fraser, 1987). At the time, job satisfaction was defined as short‐term emotional reactions resulting from satisfying needs and reducing tension, or constant feelings of a person at work and towards work (Schwab & Cummings, 1983).

It is also understood as “a pleasant or positive emotional state resulting from the assessment of our own work or the experience associated with work” (Makin, Cooper, & Cox, 2000). Job satisfaction consists of two components: cognitive (what people think about their work, to what extent they recognize their work/job as beneficial or unfavourable) and emotional (what people feel towards their work or what emotions they experience at work, their comfort at work or feelings experienced towards their work) (Jaros, 2005).

Currently, concern about the job satisfaction of nurses is growing worldwide because of their key role in determining the quality of patient care. An increase in the job satisfaction of nurses may not only ensure their appropriate employment but also improve how patients perceive the quality of their care. Predictors of job satisfaction can contribute to a comprehensive understanding of the complex phenomenon of job satisfaction, which may further help to develop efficient strategies to cope with shortages in nurses' employment and improve the quality of patient care (Lu, Zhao, & While, 2019). Employee satisfaction surveys allow to recognize the attitudes and moods prevailing among employees. Those surveys help to discover problematic areas generating conflicts and the most satisfying aspects of work (Lorber & Skela Savič, 2012).

## 2 BACKGROUND

In Poland, women work mainly as nurses (97.7%). The mean age of nurses is 52.03 years. As of the end of 2018, the total number of Registered Nurses was 333,796 and that of those employed was 233,012 (Central Register of Nurses & Midwives, 2018). The age range of nurses indicates a growing problem of generation replacement. In 2016, the rate of employed nurses in 1,000 inhabitants was 6.25. According to forecasts, in 2020, the rate will decrease to 5.67, and in 2025, there will be only 4.87 nurses/1000 inhabitants (Report of the Supreme Council of Nurses & Midwives, 2017).

Besides a family doctor and a family midwife, a family nurse (District nurse) plays a crucial role in the primary healthcare system. A patient has the right to choose them, and they are the first medical professionals the patient meets in case of a medical concern. A family nurse (also called a primary healthcare nurse) provides comprehensive nursing care over a person, family and local community. This includes the care of sick and healthy people, regardless of gender and age (excluding newborns and infants up to 2 months of age) and persons with disabilities. One nurse should have less than 2,500 patients to care about. Financing benefits in primary care is based on annual capitalization rates, based on lists of patients covered by care based on a declaration of choice (Regulation of the President of the National Health Fund, 2017).

A family nurse plans and performs nursing care of patients and their families with regard to health promotion, prophylaxis of diseases and diagnostic, therapeutic and rehabilitative benefits (The Act of 2017 October, 2017 on primary health care). The Ministry of Health describes the detailed scope of the tasks of a family nurse which include the following: advising patients on a healthy lifestyle; organizing support groups; assessing and monitoring pain; dressing wounds, bedsores and burns; and prescribing medications containing active substances and foods of special medical purposes and issuing prescriptions (Regulation of the Minister of Health, 2016).

Nursing education has few forms of professional development in Poland. We have 2 stages of study in Poland. After the study, we have 3 ways of development: specialization, qualification courses and specialist courses. Specialization—aimed at obtaining specialist qualifications in a specific field of medicine or in the field applicable in health care and the title of a specialist in this field, for example family nursing and nursing long‐term care. Qualification courses—aimed at obtaining specialist qualifications for providing specific health services falling in the scope of a given field of medicine or field applicable in health care, for example nursing teaching and upbringing and palliative care nursing. Specialist courses—aimed at obtaining qualifications to perform specific professional activities when providing health services, for example preventive vaccinations, cardiopulmonary resuscitation and electrocardiography.

A young people start study in the first stage of nursing. After three years, they have a degree of Bachelor of Nursing and are given the permit to practice their profession. Nurses can choose one of three possibilities of professional development. When they want to become practitioners of family nursing, they must have a qualification course, the second stage studies or specialization. Three specialist courses are required to start specialization: physical examination, cardiopulmonary resuscitation and electrocardiography. A nurse with a master's degree and specialization has the highest qualifications in primary health care (Regulation of the Minister of Health, 2012).

Requirements for nurses increased significantly. Nurses were promoted from support staff to the role of a partner in the therapeutic team. To perform this new role, the motivation and satisfaction with the work are necessary. There have been many studies on job satisfaction of nurses in Poland. However, these studies were conducted at the local level and there are no nationwide studies (Brayer, Foley, Doroszkiewicz, Jamiołkowski, & Marcinowicz, 2017; Kunecka, Kamińska, & Karakiewicz, 2007; Ostrowicka, Walewska‐Zielecka, & Olejniczak, 2013). Family nurses are a special group of nurses. Few researchers analysed the level and determinants of satisfaction from the work of family nurses (Czarnecka, Sienkiewicz, Kobos, Wójcik, & Krupniewicz, 2014; Walas et al., 2007).

An integrative review of papers on job satisfaction and career intentions of Registered Nurses in primary health care revealed a negative effect of poor remuneration on job satisfaction (Report of the Supreme Council of Nurses & Midwives, 2017). Other factors that were identified to negatively affect the job satisfaction of nurses include time pressure, high administrative workloads, a lack of recognition and poor role clarity. On the other hand, a professional role, respect, recognition, relationships at work and autonomy can positively affect their job satisfaction (Halcomb, Smyth, & McInnes, 2018).

*The present study* aimed to define the level of job satisfaction among Polish family nurses. Attempts were made to assess whether job satisfaction depends on seniority of this profession, location of a workplace, form of employment, family structure and financial situation.

## 3 DESIGN

A cross‐sectional study was conducted among Polish family nurses who were professionally active in 2018. The study questionnaires were sent to all (45) County Chambers of Nurses and Midwives in Poland. Five questionnaires were sent to each County Chamber of Nurses and Midwives, and they were requested to pass them along to a minimum of five family nurses who wish to respond to the questionnaire. To increase the return rate of questionnaires, the researcher (co‐author of the study) contacted each chamber by phone and requested to complete and return the questionnaires. Questionnaires were sent to a total of 225 participants, of which 220 correctly completed and returned the questionnaires (97.8%).

## 4 METHODS

### 4.1 Collection of data

Two questionnaires were sent to the participants for collecting data: the standardized questionnaire “The Satisfaction with Job Scale” developed by A.M. Zalewska (2003) and a questionnaire prepared by us for the purpose of this study.

### 4.2 “The Satisfaction with Job Scale”

“The Satisfaction with Job Scale” is an accurate and reliable research tool, which enables measuring the cognitive aspect of overall job satisfaction. It contains five questions with possible answers on the scale from 1–7:1—strongly disagree, 2—disagree, 3—rather disagree, 4—it is difficult to say whether I agree or disagree, 5—rather agree, 6—agree and 7—strongly agree; the higher the score, the higher is the level of job satisfaction (Zalewska, 2003).

The Job Satisfaction Scale is a valuable, reliable and accurate tool for measuring overall job satisfaction. This study is inspired by The Satisfaction With Life Scale (SWLS) technique by Diener, Emmons, Larsen, and Griffin (1985) and designed to measure overall life satisfaction. Following the above technique, the Job Satisfaction Scale was constructed (Zalewska, 2001). All the statements are elements of one dimension and show high internal consistency (Cronbach's alpha coefficient above 0.80) in a heterogeneous sample of employees. The advantage of the technique is the fact that the assessment of work is made by employees based on their personal criteria. Cronbach's alpha coefficient in our research was 0.85.

### 4.3 The author's survey questionnaire

The survey questionnaire prepared for this study consists of 30 questions (27 closed‐ended and 3 open‐ended). The closed‐ended questions were related to the demographic data of the family nurses, such as their sex, age, education and the place of residence, as well as their family, financial and professional situation, while the open‐ended questions asked the respondents to indicate the reasons of satisfaction and dissatisfaction with job and to report additional comments (if any).

### 4.4 Analysis

The collected data were encoded and analysed using the Statistica 13.1 version software package (StatSoft) (StatSoft, 2006). The data were presented as mean and standard deviation (*SD*) or as numbers. The normality of distribution was determined using the Shapiro–Wilk test. In addition, Mann–Whitney *U* test, ANOVA multiple range test, Kruskal–Wallis test and post hoc multiple comparisons with mean ranks were used for the statistical analysis. Results with a *p*‐value of <.05 were considered as statistically significant.

### 4.5 Ethics

The study was approved by the Ethics Committee of the Medical University of Bialystok, Poland (no. R‐I‐002/413/2017). The consent obtained from the participants was verbal, and the completion of our survey was considered implied consent to participate. Informed consent was implied by submission of a completed survey questionnaire.

## 5 RESULTS

### 5.1 Characteristics of respondents

Of the 220 family nurses surveyed, 219 (99.5%) were females and one (0.5%) was male. The average age of the nurses was 50.13 years (*SD* 8.36). Most nurses had secondary education (37.6%), a qualification course (96.2%), a job seniority of 30–39 years (44.6%) and were residing in a medium‐sized city (43.7%). Only 39% of them had a specialization. The average age of nurses in Poland is 52.6 years. A higher percentage of men (2.5%) is observed in the general population of nurses. The percentage distribution of nurses with secondary education significantly differed in our studies in relation to the general population of nurses (37.6% vs. 74.2%) (Main Council of Nurses and Midwives, 2018) (Table 1).

**TABLE 1:** Characteristics of respondents and the entire population of nurses in Poland

	Study participants *N = *220 (100%)	The entire population of nurses^a^ *N* = 299,619 (100%)
Age (years), Mean	50.13	52,59
Sex		
Female	219 (99.6%)	292,437 (97.5%)
Male	1 (0.45%)	7,182 (2.5%)
Education		
Secondary (Secondary school/medical secondary school)	80 (37.6%)	219,217 (74.2 %)
**Higher (Bachelor's degree)**	66 (31.0%)	50,077 (17.0%)
**Higher (Master of nursing science or other)**	67 (31.5%)	26,143 (8.9%)

^a^ The number of nurses and midwives registered and employed in Poland (Data from the Supreme Chamber of Nurses and Midwives).

### 5.2 Job satisfaction based on “The Satisfaction with Job Scale”

The mean score of job satisfaction of the study group was 22.23 (on a scale of 35), and the median score (Me) was 23; the lower quartile (Q1) was 18, while the upper quartile (Q3) was 26.

### 5.3 Job satisfaction and seniority in the nurse's profession

**FIGURE 1:**
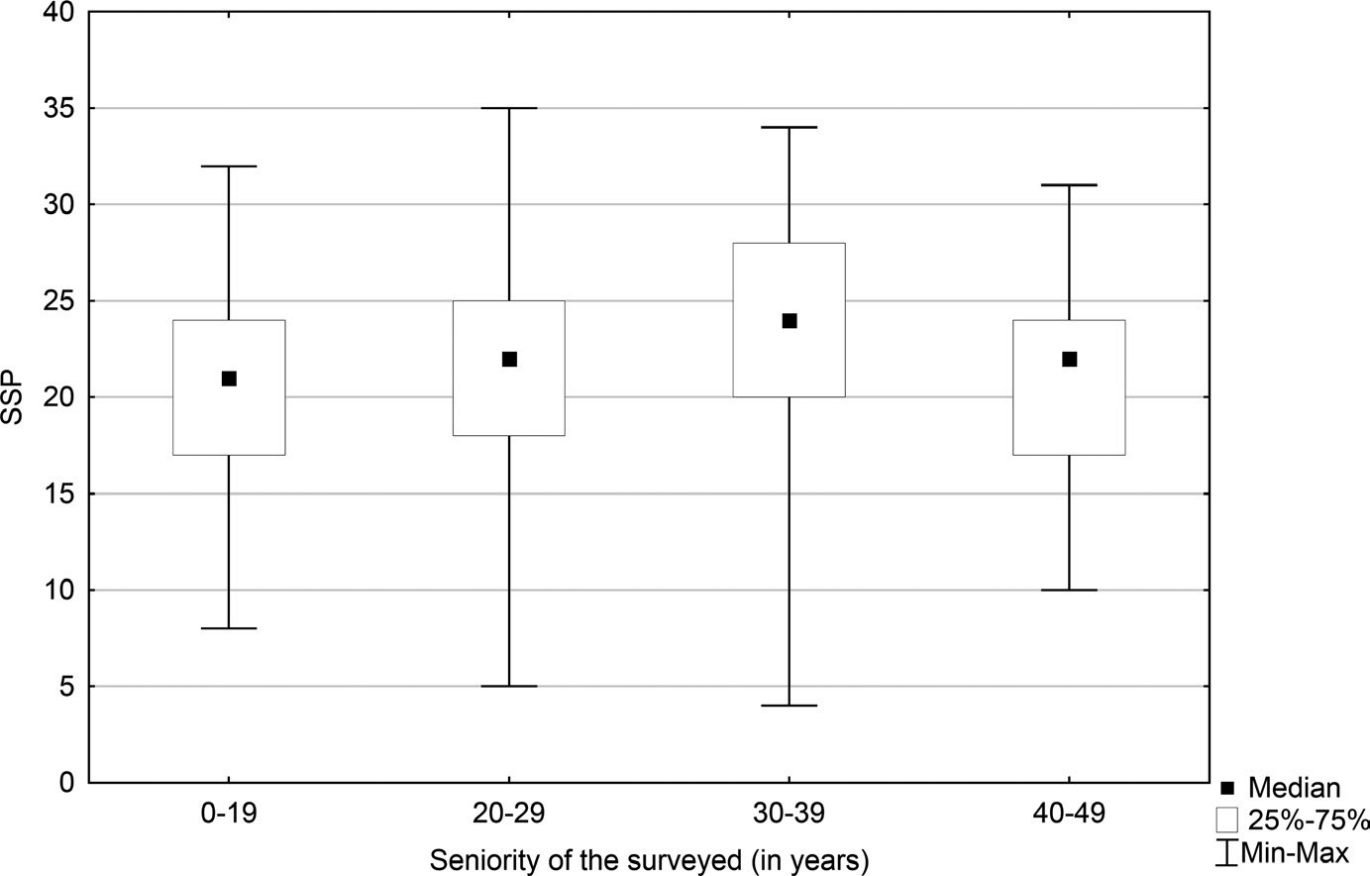
Relationship between job satisfaction level and the seniority in the nurse profession

A statistically significant difference at the level (*p* = .039) was found between the level of job satisfaction and the seniority in the profession of a nurse. The median of the job satisfaction level for the respondents of up to 19‐year seniority was 21 scores, for the people with 20‐ to 29‐year seniority reached 22 scores, for the nurses with 30–39 years on the job—24 scores and for the professionals with above 40‐year seniority—22 scores. Nurses with the seniority of 30–39 years (24 scores) were the most satisfied with a job, while nurses of the seniority up 19 years (21 scores) were the least satisfied with a job. The difference was statistically significant (*p* = .032) (Figure 1).

### 5.4 Job satisfaction and the workplace

**FIGURE 2:**
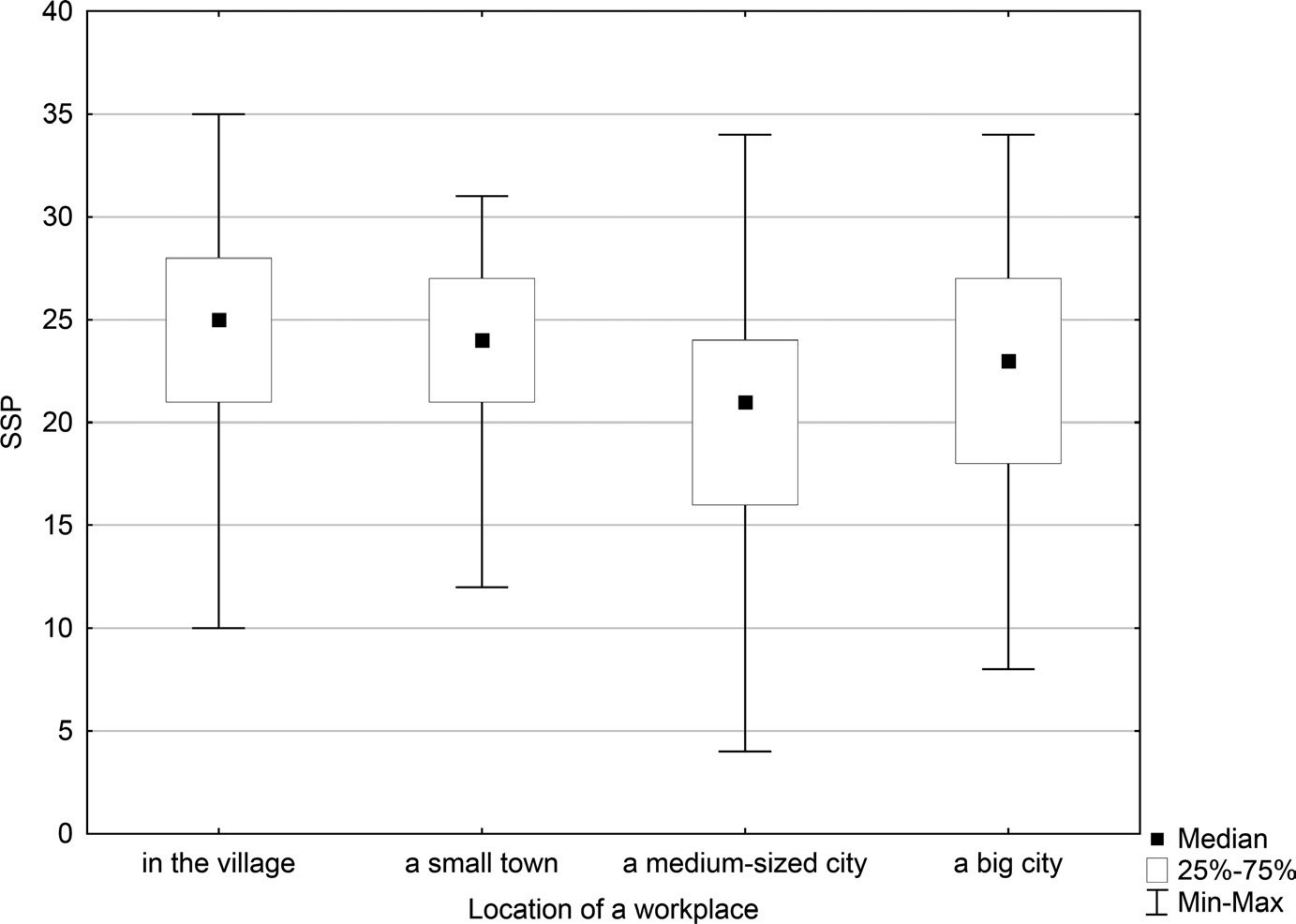
Relationship between the job satisfaction level and the workplace

The statistically significant relationship was found between job satisfaction and the workplace of the respondents (*p* < .003). The median of job satisfaction among nurses working in the village totalled 25 scores, in a small town—24 scores and in a medium‐sized city—21 scores, while in a big city—23 scores. In addition, a statistically significant difference in job satisfaction was found between the nurses working in the village and those working in a medium‐sized city (*p* = .010), as well as between the nurses working in a small town and those working in a medium‐sized city (*p* = .047). Professionals with the highest level of job satisfaction were working in the village, whereas the lowest level of job satisfaction was reported by those working in medium‐sized cities (Figure 2).

### 5.5 Job satisfaction and the form of employment

**FIGURE 3:**
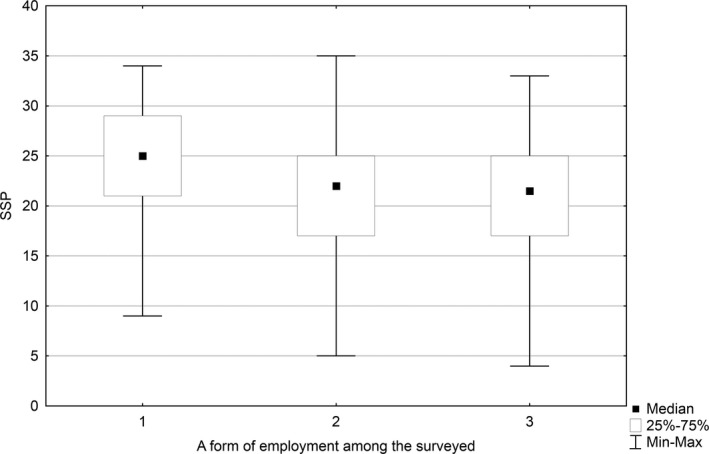
Relationship between the job satisfaction level and the form of employment (1—owners or co‐owners of a primary healthcare unit, 2—employees on a full‐time contract and 3—employees on a half‐time contract or on other types of a contract

A statistically significant difference was found between the level of job satisfaction and the form of nurses' employment (*p* = .001). The median of job satisfaction determined for owners and co‐owners of a primary healthcare unit was 25 scores, while that for the nurses employed on a full‐time contract and those employed on a half‐time contract or on other types of contract was 22 scores. The level of job satisfaction was statistically significantly higher among nurses who are owners or co‐owners of a primary healthcare unit than in those employed on a full‐time contract (*p* = .001) and those employed on half‐time contract or on other types of contract (*p* = .045) (Figure 3).

### 5.6 Job satisfaction and family structure

**FIGURE 4:**
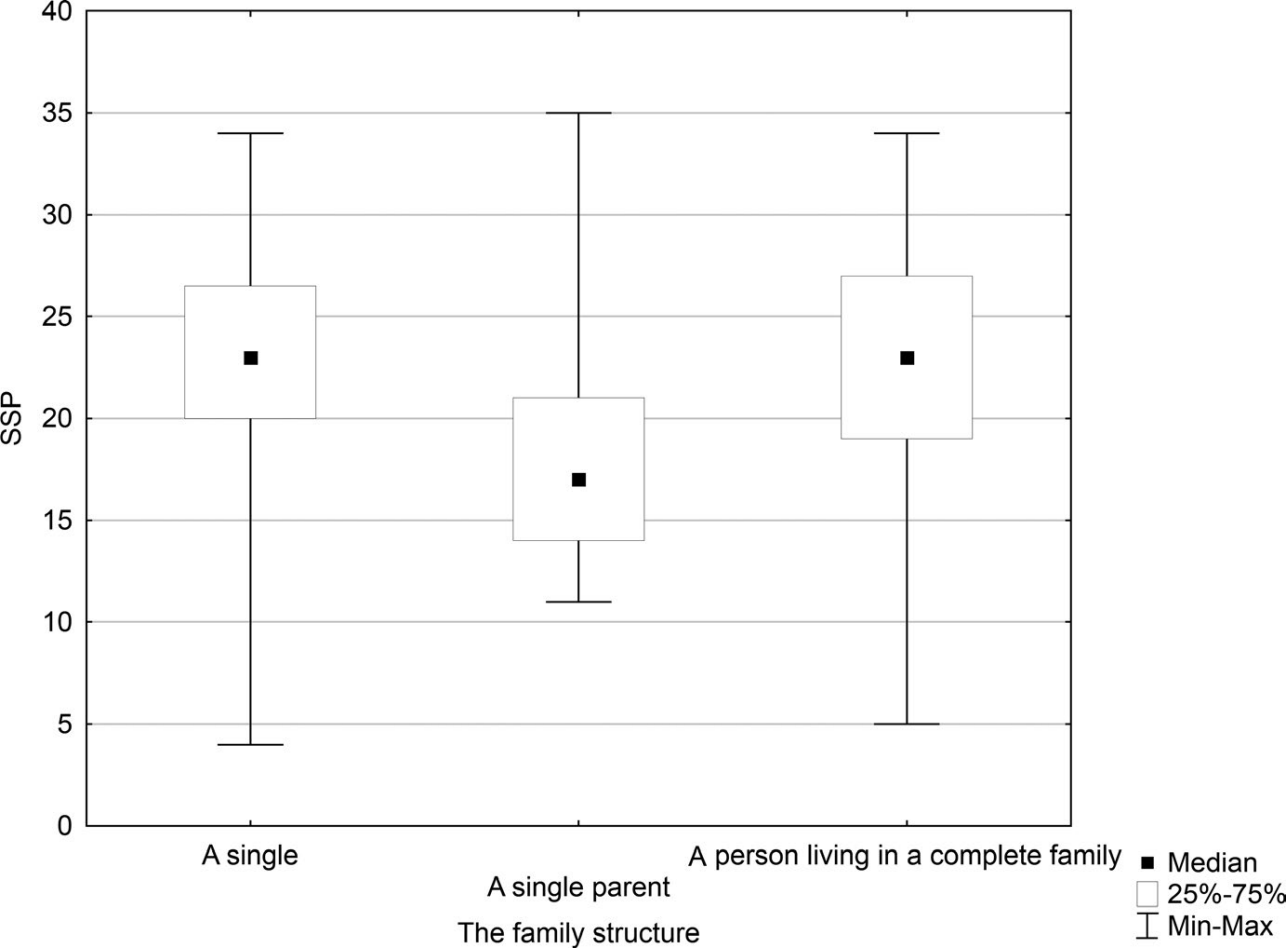
Relationship between the level of job satisfaction and the structure of a family

A statistically significant relationship (*p* = .007) was established between the level of job satisfaction and the structure of family declared by the respondents. The median of the level of job satisfaction was 23 for singles, 17 for professionals who were single parents and 23 for the nurses living in a complete family. Based on the results of multiple comparisons test using mean ranks, Kruskal–Wallis test was performed for the groups that differed statistically significantly: “single” versus “single parent” (*p* = .022) and “single parent” versus “complete family” (*p* = .005). Thus, the level of job satisfaction was the highest among nurses living in a complete family and those who are single (Figure 4).

### 5.7 Job satisfaction level and the status and financial situation of the study participants

Most respondents declared their financial status as very good or good, and the median of the level of job satisfaction for these participants was 29 score. On the other hand, professionals who declared their financial status as average or bad had a lower level of job satisfaction (Me = 21) and the difference was considered statistically significant (*p* < .001). Furthermore, a significantly higher level of job satisfaction was found among the professionals who declared that they could afford to buy what they wanted and possessed savings (Me = 29) compared with those who could only afford to buy necessities (Me = 22; *p* < .001; Table 2).

**TABLE 2:** Level of job satisfaction with regard to the respondents' financial status and situation

	Me (Q_1_;Q_3_)	*N*	*p* ^a^
Financial status of a respondent's family			
Very good/good	29 (27;31.5)	139	<.001
Average/bad	21 (16;24)	74	
Respondents' financial situation			
I can afford to buy what is necessary but not everything	22 (17;25)	190	<.001
I can afford to buy everything I want	29 (22;31)	23	

^a^ Mann–Whitney test.

### 5.8 Level of job satisfaction with regard to participation in scientific conferences and subscription of nursing care journals

A total of 29.1% of respondents declared their participation in a scientific conference on nursing and health care during the last 2 years. These respondents had a significantly higher level of job satisfaction than those who did not participate in any scientific conferences on nursing and health care (*p* = .032). The median of the level of job satisfaction determined for those respondents was 24 and 22 score.

A total of 28.2% of respondents declared having subscribed to nursing care journals (*p* = .016). These respondents had a higher level of job satisfaction compared with those not subscribing to any nursing care journals. The median of the level of job satisfaction was 24 and 22 score (Table 3).

**TABLE 3:** Summary of the level of job satisfaction with regard to participation in a scientific conference and subscription of nursing care journals

	Level of job satisfaction				
	Yes		No		*p* ^a^
	Me (Q_1_;Q_3_)	*N* (%)	Me (Q_1_;Q_3_)	*N* (%)	
Participation in a scientific conference on nursing and health care during the last 2 years	24 (21;27)	62 (29.1)	22 (17;26)	151 (70.9)	.032
Subscription of nursing care journals	24 (21;27)	60 (28.2)	22 (17;26)	153 (71.8)	.016

^a^ Mann–Whitney test.

### 5.9 Level of satisfaction and recommendation to work in a primary healthcare unit to other nurses

**FIGURE 5:**
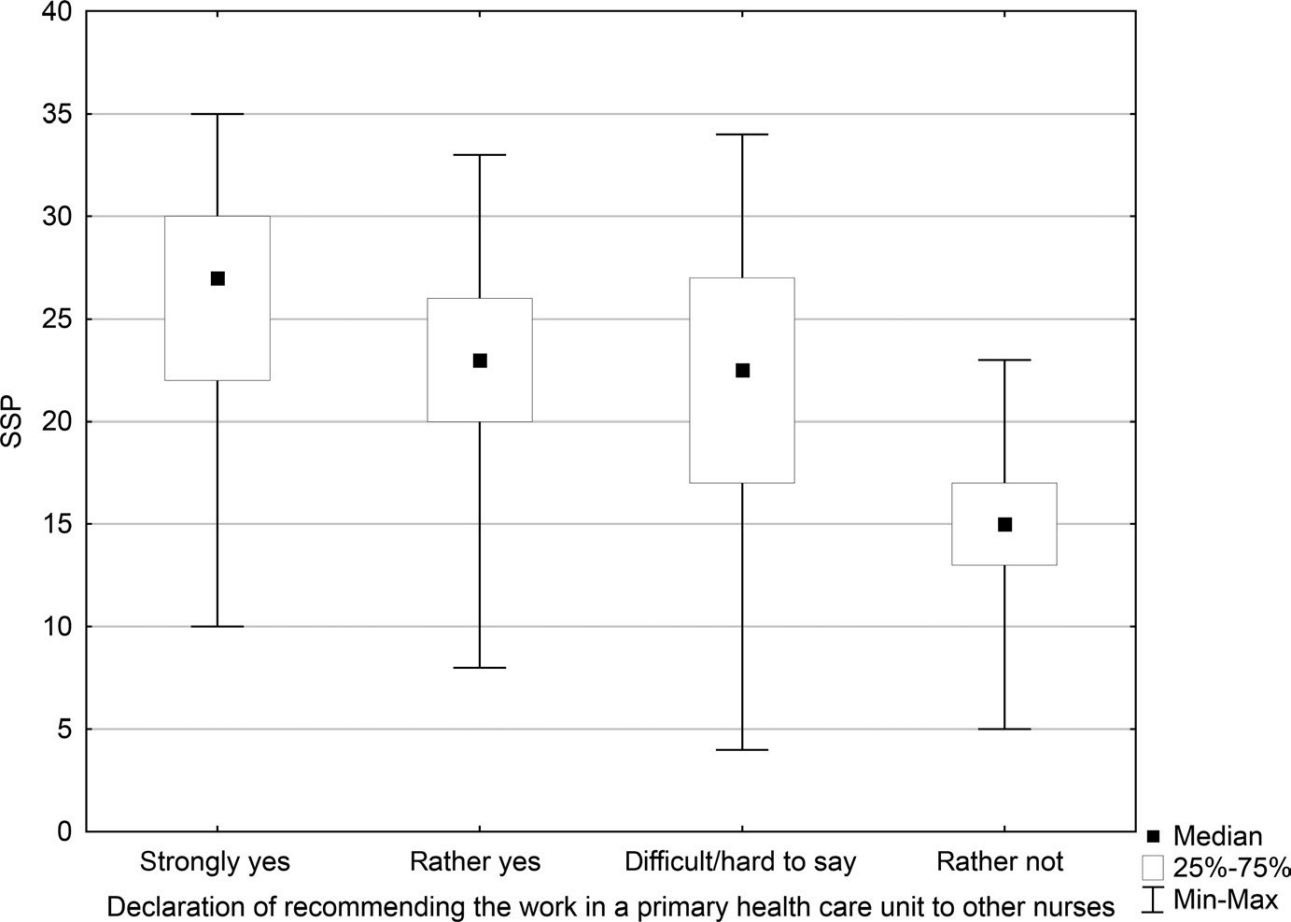
Relationship between the level of job satisfaction and the recommendation of work in a primary healthcare unit to other nurses

A statistically significant relationship was established between the level of job satisfaction and the declaration of recommending work in a primary healthcare unit to other nurses (*p* < .001). The median of the level of job satisfaction for the answer “strongly yes” was 27, while that for the answer “rather yes” was 23, for the answer “difficult to say” was 22.5 and for the answer “rather not” was 15. None of the respondents chose the option “strongly no.” The highest level of job satisfaction was reported by the nurses who strongly recommended the work in primary healthcare units to other nurses, while the lowest was reported by those who rather did not recommend the work in primary healthcare units (Figure 5).

### 5.10 Causes of job satisfaction and dissatisfaction

The participants' responses to the closed‐ended question whether you are satisfied with work as a family nurse, on a 5‐degree scale, were as follows: very satisfied (23.9%), rather satisfied (60.6%), neither satisfied nor dissatisfied (11.3%), rather dissatisfied (3.3%) and very dissatisfied (0.9%).

Additionally, the surveyed participants were asked two open‐ended questions: “What makes you satisfied with a job of a family nurse?” and “What makes you dissatisfied with a job of a family nurse?”

The total number of answers received for the open‐ended questions referring to the causes of job satisfaction was 252, while that received for the questions about the causes of dissatisfaction was 140 (Table 4).

**TABLE 4:** Causes of a family nurse's satisfaction or dissatisfaction with a job—categories of answers to open‐ended questions

Cause of satisfaction	*N* = 252	Number of respondents (%)
Close contact with patients	81	32.1
Professional independence	77	30.6
Own work organization	25	9.9
High self‐satisfaction and a greater possibility of additional training	58	23.0
No night shifts	11	4.4

Causes of dissatisfaction	*N* = 140	
Low salary	43	30.7
Psychic burden with patients' problems	22	15.7
No co‐operation from a patient or his/her family	19	13.6
Underestimation of a nurse's job by co‐workers and patients	18	12.9
Difficulty of regulations referring to a nurse's job	17	12.1
Abundance of medical documentation	16	11.4
High number of patients under a nurse's care	5	3.6

Statements referring to job satisfaction provided by the participants were as follows: “frequent contact with little children, satisfaction with helping the elderly and patient's gratitude,” “greater independence, care of a patient and a family and community awareness,” “contact with people and independent organization of work,” “work conditions and atmosphere at work, possibility of additional trainings and no monotony,” “continuity of a patient's care by one person—a nurse” and “no night shifts.”

Statements referring to job dissatisfaction provided by the participants were as follows: “financial status and frequent patients' demands,” “low salary and much bureaucracy,” “overload with administrative work,” “abundance of medical documentation and risk and stress connected with making decisions about threats to a patient's health status” and “some patients claim about staff shortages in relation to the number of patients and their needs and frequent changes in nursing job regulations and difficulties in their understanding.”

## 6 DISCUSSION

Research on the professional satisfaction of nurses seems to be very important because they are a large professional group of growing importance in the ageing society in Poland. The latest data show that the population of nurses and midwives is ageing rapidly. The average age of a nurse in 2008 was 44.19, and in 2019, it already reached 52.59 years (The Supreme Chamber of Nurses & Midwives, 2018).

In our study, a statistically significant relationship was found between the level of job satisfaction and the seniority in the nurse profession, location of the respondents' workplace, the form of employment, the family structure, the financial status and situation, participation in scientific conferences and subscription of nursing care journals.

A Polish study conducted among 189 nurses reported that the level of job satisfaction increases together with the respondents' age (Kosińska & Pilarz, 2005). The results of our study are in good agreement with the reports in the literature that older nurses are more satisfied with the job than younger ones. Additionally, the study by Lorber and Savič analysed the level of job satisfaction among nurses in the Slovenian hospitals and the factors affecting their job satisfaction and confirmed the correlation between the seniority and the level of job satisfaction (Lorber & Savič, 2012). Our study also showed that the level of job satisfaction changed with the seniority of nurses. The respondents with shorter seniority were less satisfied with their job, whereas those with higher seniority reported more satisfaction and the level of satisfaction peaked with the seniority of 30–39 years in this profession. However, the level of job satisfaction decreased among nurses working for more than 40 years. This may be associated with the burnout syndrome.

In our study, nurses working in the village had the highest level of job satisfaction, whereas those working in medium‐sized cities had the lowest level of job satisfaction. However, in other studies, no statistically significant relationships were found between the place of residence and the level of job satisfaction (Wysokiński et al., 2009).

Family nurses in Poland can also work in various organizational and legal forms. The most common form of employment is contracting nursing services by a doctor who hires a nurse. Another form, used more rarely, is founding healthcare units by nurses and signing up the contract with National Health Fund. Interestingly, in our study, the level of job satisfaction was markedly higher among those nurses who were the owners or co‐owners of a healthcare unit than among those who were employed. Greater professional independence contributes to satisfaction with a job, which was also confirmed by the results reported in the literature (Curtis & Glacken, 2014).

Furthermore, in our study, a statistically significant relationship was found between the level of job satisfaction and the family structure declared by the respondents. The highest level of job satisfaction was reported by the nurses living in a complete family and as singles compared with those who were single parents. In a previous study conducted among retired nurses, it was shown that marital status influenced significantly the level of job satisfaction and the nurses being in a relationship were more satisfied with their job than those who were single (Krzos, Charzyńska‐Gula, Stanisławek, Szadowska‐Szlachetka, & Rząca, 2014).

Some studies also showed the relationship between the level of job satisfaction and the financial situation of nurses working in Poland (Andruszkiewicz, 2007; Kunecka, 2010) and in other countries (Delobelle et al., 2011). The results of these studies were in line with our study which confirmed that the nurses who declared an average or poor financial status had a lower level of job satisfaction.

Additionally, the respondents declaring that they could afford to buy everything that they wanted and had savings had a higher level of job satisfaction than those who could only afford to buy the necessary articles. In addition, the results of the study showed that the nurses who declared their participation in scientific conferences and subscription of nursing care journals had a markedly higher level of job satisfaction than those not participating in scientific conferences and not subscribing to nursing care journals. In a study by Łagun, job satisfaction was found to have a significant relationship with the chances of achieving a goal, which is to take up training, as well as the assessment of the training value. The employees who were satisfied with their job assessed positively the goal, which was to undertake training and developmental activities (Łaguna, 2006).

Recommending the work to other nurses had a significant relationship with the level of job satisfaction. The nurses characterized by a high level of job satisfaction more frequently recommended the work to other nurses. Numerous studies have confirmed that higher salary, greater professional autonomy, wider possibilities of upgrading qualifications and improving work conditions are some factors that positively affect job satisfaction (Best & Thurston, 2006; Curtisma & Glacken, 2014; Tourangeau et al., 2014). Similarly, in our study, a statistically significant relationship was observed between the level of job satisfaction and declaration of recommending the work in a primary healthcare unit to other nurses. The nurses who strongly recommended the work in a primary healthcare unit to other nurses had the highest level of job satisfaction compared with those who did not recommend such work.

Nursing job is regarded as one of the social professions, and a low level of job satisfaction is one of the main causes of occupational burnout (Dębska & Cepuch, 2008). In our study, the respondents indicated, among others, independence in the work, own organization of work, high self‐satisfaction and no night shifts as the causes of job satisfaction of nurses working in a primary healthcare unit. The surveyed respondents mentioned a psychic burden with patients' problems, non‐co‐operation of a patient or his/her family, low salary and the abundance of medical documentation as the causes of job dissatisfaction. Gawęda, Śnieżek, and Serzysko (2018) presented similar results in their study which showed that shortage of nurses in relation to the number of patients and their health status, low salary and high psychic and physical burden influenced job satisfaction. Another study confirmed that the relationships one had with other people at work were also one of the factors affecting satisfaction with the job (Storey, Cheater, Ford, & Leese, 2009).

The main strength of this study is that the data were collected from family nurses employed in various primary healthcare facilities throughout Poland. High response rates contribute to the external validity. However, no information is available about the nurses who chose not to participate in the present study and it is possible that they have a different view of the questions asked. The limitation of the study is the fact that individual District Chamber of Nurses and Midwives (OIPiP) were freedom to distribution of questionnaires surveys in their area. Future studies of this kind could increase the sample size for the study to have higher external validity.

## 7 CONCLUSION

Our study showed that the Polish family nurses are moderately satisfied with their job. A higher level of job satisfaction was reported by those family nurses with longer job seniority, those who were working in the country, those who were owners or co‐owners of a primary healthcare unit, those who were living in a complete family or as single and those who could afford to buy what they wanted and possessed savings. Moreover, the level of job satisfaction was the highest among those nurses who recommended the work in a primary healthcare unit to other nurses, participated in scientific conferences and subscribed to nursing care journals. The causes of job satisfaction reported in the study were, among others, independence in the work, own organization of work, high self‐satisfaction and no night shifts, while psychic burden with patients' problems, lack of co‐operation with a patient or his/her family, low salary and abundance of medical documentation were reported as the causes of job dissatisfaction among the family nurses.

The practical conclusion of the study is the suggestion that management should influence the psychological climate in the workplace. Positive assessment of climate by the employee will make him more satisfied with his work. Furthermore, managers of primary care facilities should know that to increase the level of satisfaction of nurses with their professional activities, they must increase the sense of meaning and independence of work. Additionally, an increase in pay would contribute to increased interest in the work of a family nurse. The previous section mentioned the limitations of our study. To present the level of job satisfaction in the general population of nurses, it would be necessary to repeat the survey among nurses from various workplaces.

## CONFLICT OF INTEREST

The author(s) declare that they have no competing interests.

## AUTHORS' CONTRIBUTIONS

All authors fulfil the criteria of authorship according to the Vancouver rules for authorship. PK and LM drafted this paper in close co‐operation. All authors were involved in interpretation of the results and editing of the manuscript. The study was designed by PK and LM. All authors read and approved the final manuscript.

## References

[nop2550-bib-0001] Andruszkiewicz, A. (2007). Types of behaviors and experience connected with work in the nurses group. Nursing Topics, 15(2–3), 159–160.

[nop2550-bib-0002] Bańka, A. (2005). Organizational psychology In Strelau J. (Ed.), Psychology. Academic handbook (vol. 3, pp. 321–350). Gdańsk, Poland: Gdansk Psychological Publishing House.

[nop2550-bib-0003] Best, M. F. , & Thurston, N. E. (2006). Canadian public health nurses' job satisfaction. Public Health Nursing, 23(3), 250–255. 10.1111/j.1525-1446.2006.230307.x 16684203

[nop2550-bib-0004] Brayer, A. , Foley, M. , Doroszkiewicz, H. , Jamiołkowski, J. , & Marcinowicz, L. (2017). Job satisfaction among masters in nursing in Central and East Poland: A preliminary study. Family Medicine & Primary Care Review, 19(1), 7–11. 10.5114/fmpcr.2017.65083

[nop2550-bib-0005] Brief, A. P. , & Roberson, L. (1989). Job attitude organization: An exploratory study. Journal of Applied Social Psychology, 19, 717–727. 10.1111/j.1559-1816.1989.tb01254.x

[nop2550-bib-0006] Central Register of Nurses and Midwives . (2018). Retrieved from https://nipip.pl/liczba‐pielegniarek‐poloznych‐zarejestrowanych‐zatrudnionych/

[nop2550-bib-0007] Curtis, E. A. , & Glacken, M. (2014). Job satisfaction among public health nurses: A national survey. Journal of Nursing Management, 22(5), 653–663. 10.1111/jonm.12026 25041804

[nop2550-bib-0008] Curtisma, E. A. , & Glacken, M. (2014). Job satisfaction among public health nurses: A national survey. Journal of Nursing Management, 22, 653–663. 10.1111/jonm.12026 25041804

[nop2550-bib-0009] Czarnecka, J. , Sienkiewicz, Z. , Kobos, E. , Wójcik, G. , & Krupniewicz, A. (2014). Risks associated with the work of district nurses. Polish Nursing, 4(54), 296–301.

[nop2550-bib-0010] Dębska, G. , & Cepuch, G. (2008). Professional burnout in nurses working in the primary health care. Nursing Topics, 16(3), 273–279.

[nop2550-bib-0011] Delobelle, P. , Rawlinson, J. L. , Ntuli, S. , Malatsi, I. , Decock, R. , & Depoorter, A. M. (2011). Job satisfaction and turnover intent of primary healthcare nurses in rural South Africa: A questionnaire survey. Journal of Advanced Nursing, 67(2), 371–383. 10.1111/j.1365-2648.2010.05496.x 21044134

[nop2550-bib-0012] Diener, E. , Emmons, R. A. , Larsen, R. J. , & Griffin, S. (1985). The satisfaction with life scale. Journal of Personality Assessment, 49(1), 71–75. 10.1207/s15327752jpa4901_13 16367493

[nop2550-bib-0013] Fraser, T. M. (1987). Human stress, work and job satisfaction. A critical approach. Geneva, Switzerland: International Labour Office.

[nop2550-bib-0014] Gawęda, A. , Śnieżek, A. , & Serzysko, B. (2018). Job satisfaction in the opinion of surveyed nurses. Nursing and Public Health, 8(4), 269–276. 10.17219/pzp/91608

[nop2550-bib-0015] Halcomb, E. , Smyth, E. , & McInnes, S. (2018). Job satisfaction and career intentions of registered nurses in primary health care: An integrative review. BMC Family Practice, 19, 136 10.1186/s12875-018-0819-1 30086722 PMC6081816

[nop2550-bib-0016] Hersey, R. B. (1932). Workers' emotions in shop and home: A study of individual workers from the psychological and physiological standpoint. Philadelphia, PA: University of Pennsylvania Press.

[nop2550-bib-0044] Hoppock, R. (1935). Job Satisfaction. New York and London: Harper and Brothers.

[nop2550-bib-0017] Jaros, R. (2005). Zadowolenie z pracy In Golińska L. (Ed.), Skuteczniej, sprawniej, z większą satysfakcją (pp. 85–105). Lodz, Poland: Wydawnictwo Naukowe Wyższej Szkoły Kupieckiej Łódź.

[nop2550-bib-0018] Kosińska, M. , & Pilarz, Z. (2005). Satysfakcja pielęgniarek z pracy zawodowej i jej uwarunkowania. Annales Universitatis Mariae Curie‐Skłodowska Lublin, 60(16), 236.

[nop2550-bib-0019] Krzos, A. , Charzyńska‐Gula, M. , Stanisławek, A. , Szadowska‐Szlachetka, Z. , & Rząca, M. (2014). The analysis of factors influencing the presence or lack of job satisfaction among nurses at the end of their career. Journal of Health Sciences, 4(5), 11–24.

[nop2550-bib-0020] Kunecka, D. (2010). Employee's satisfaction and medical service quality. Problemy Higieny I Epidemiologii, 91(3), 451–457.

[nop2550-bib-0021] Kunecka, D. , Kamińska, M. , & Karakiewicz, B. (2007). Analysis of factors determining job satisfaction among nurses. Preliminary Research. Nursing Problems, 15, 192–196.

[nop2550-bib-0022] Łaguna, M. (2006). Satysfakcja z życia i satysfakcja z pracy a motywacja do podejmowania szkoleń: Doniesienie z badań. Psychology of Quality of Life, 11(2), 163–172. 10.5604/16441796.1058440

[nop2550-bib-0045] Locke, E. A. (1976). The Nature and Causes of Job Satisfaction. Handbook of Industrial and Organizational Psychology, 1, 1297–1343.

[nop2550-bib-0023] Lorber, M. , & Savič, B. S. (2012). Job satisfaction of nurses and identifying factors of job satisfaction in Slovenian Hospitals. Croatian Medical Journal, 53(3), 263–270. 10.3325/cmj.2012.53.263 22661140 PMC3368291

[nop2550-bib-0024] Lorber, M. , & Skela Savič, B. (2012). Job satisfaction of nurses and identifying factors of job satisfaction in Slovenian Hospitals. Croatian Medical Journal, 53(3), 263–270. 10.3325/cmj.2012.53.263 22661140 PMC3368291

[nop2550-bib-0025] Lu, H. , Zhao, Y. , & While, A. (2019). Job satisfaction among hospital nurses: A literature review. International Journal of Nursing Studies, 94, 21–31. 10.1016/j.ijnurstu.2019.01.011 30928718

[nop2550-bib-0026] Main Council of Nurses and Midwives (2018). Number of nurses and midwives registered and employed. Retrieved from https://nipip.pl/liczba‐pielegniarek‐poloznych‐zarejestrowanych‐zatrudnionych/

[nop2550-bib-0027] Makin, P. J. , Cooper, C. L. , & Cox, C. H. (2000). Organizacje a kontrakt psychologiczny: zarządzanie ludźmi w pracy (p. 82). Warszawa, Poland: PWN Wydawnictwo Naukowe.

[nop2550-bib-0028] Mayo, E. (1933). The human problems of an industrial civilization. New York, NY: Macmillan Co.

[nop2550-bib-0029] Ostrowicka, M. , Walewska‐Zielecka, B. , & Olejniczak, D. (2013). Motivation in nurse's work and job satisfaction. Public Health and Governance, 11(2), 191–209. 10.4467/20842627OZ.14.017.1627

[nop2550-bib-0030] Regulation of the Minister of Health (2012). Regulation of the Minister of Health of August 20, 2012 regarding detailed requirements for the education of nurses and midwives 2012 item 631. Retrieved from http://prawo.sejm.gov.pl/isap.nsf/DocDetails.xsp?id=WDU20120000631

[nop2550-bib-0031] Regulation of the Minister of Health (2016). Regulation of the Minister of Health of September 21, 2016 on the scope of tasks of the primary care physician, primary care nurse and midwife of primary health care. Journal of Laws of the Republic of Poland, item 1567. Retrieved from http://prawo.sejm.gov.pl/isap.nsf/download.xsp/WDU20160001567/O/D20161567.pdf

[nop2550-bib-0032] Regulation of the President of the National Health Fund (2017). Regulation of the President of the National Health Fund regarding the conditions for the conclusion and implementation of contracts for the provision of healthcare services in the field of primary healthcare. Retrieved from https://www.nfz.gov.pl/zarzadzenia‐prezesa/zarzadzenia‐prezesa‐nfz/zarzadzenie‐nr‐1222017dsoz,6699.html

[nop2550-bib-0033] Report of the Supreme Council of Nurses and Midwives (2017). Securing Polish society in the provision of nurses and midwives (2nd ed., pp. 34–36). Retrieved from https://nipip.pl/wp‐content/uploads/2017/03/Raport_druk_2017.pdf

[nop2550-bib-0034] Schwab, D. P. , & Cummings, L. L. (1983). Review of theories regarding the relationship between task performance and satisfaction (pp. 184–198). Warszawa, Poland: Human Behavior in Organization PWN.

[nop2550-bib-0046] Spector, P. E. (1997). Job Satisfaction. Thousand Oaks, CA: SAGE Publications.

[nop2550-bib-0035] StatSoft (2006). Elektroniczny Podręcznik Statystyki PL, Krakow. Retrieved from http://www.statsoft.pl/textbook/stathome.html

[nop2550-bib-0036] Storey, C. , Cheater, F. , Ford, J. , & Leese, B. (2009). Retaining older nurses in primary care and the community. Journal of Advanced Nursing, 65(7), 1400–1411. 10.1111/j.1365-2648.2009.05009.x 19457002

[nop2550-bib-0037] The Act of 27 October 2017 on primary health care (2017). Journal of Laws, item 2217. Retrieved from http://prawo.sejm.gov.pl/isap.nsf/download.xsp/WDU20170002217/T/D20172217L.pdf

[nop2550-bib-0038] The Supreme Chamber of Nurses and Midwives (2018). Number of nurses and midwives registered and employed in Poland. Retrieved from https://nipip.pl/liczba‐pielegniarek‐poloznych‐zarejestrowanych‐zatrudnionych/

[nop2550-bib-0039] Tourangeau, A. , Patterson, E. , Rowe, A. , Saari, M. , Thomson, H. , Macdonald, G. , … Squires, M. (2014). Factors influencing home care nurse intention to remain employed. Journal of Nursing Management, 22, 1015–1026. 10.1111/jonm.12104 23905629

[nop2550-bib-0040] Walas, L. , Kachaniuk, H. , Kusiak, L. , Fidecki, W. , Sadurska, A. , & Wysokiński, M. (2007). Professional satisfaction of community/family nurses. Nursing and Public Health, 117(1), 32–35.

[nop2550-bib-0041] Wysokiński, M. , Fidecki, W. , Walas, L. , Ślusarz, R. , Sienkiewicz, Z. , Sadurska, A. , & Kachaniuk, H. (2009). Polish nurses' satisfaction with life. Nursing Problems, 17(3), 167–172.

[nop2550-bib-0042] Zalewska, A. (2001). “Work Description Inventory” O. Neubergera i M. Allerbeck – Adaptation to Polish conditions. Psychological Studies, 39(1), 197–217.

[nop2550-bib-0043] Zalewska, A. M. (2003). The Satisfaction with Job Scale – A measure of cognitive aspect of overall job satisfaction. Acta Universitatis Lodziensis, Folia Psychologica, 7, 49–61.

